# To Improve Motivational Barriers to Retention in High Resolution Anoscopy, Patients and Providers Recommend Social and Environmental Changes: A Sequential Explanatory Mixed-Methods Pilot Study in a Federally Qualified Health Center

**DOI:** 10.1007/s10461-024-04419-7

**Published:** 2024-07-12

**Authors:** Bryan A. Kutner, Baichun Hou, Rebecca Giguere, Will DeWitt, Mei Tan, Christine Tagliaferri Rael, Asa Radix, Theodorus G. M. Sandfort

**Affiliations:** 1 Department of Psychiatry and Behavioral Sciences, Albert Einstein College of Medicine and Montefiore Medical Center, Psychiatry Research Institute at Montefiore Einstein (PRIME), 1225 Morris Park Ave., Suite 4A, Bronx, NY 10461 USA; 2 HIV Center for Clinical & Behavioral Studies at New York State Psychiatric Institute and Columbia University, New York, NY USA; 3 Open Door Health, Providence, RI USA; 4 Callen-Lorde Community Health Center, New York, NY USA; 5 College of Nursing, University of Colorado, Aurora, CO USA; 6 Columbia University Mailman School of Public Health, New York, NY USA

**Keywords:** Anal cancer prevention, High resolution anoscopy, COM-B Model, Behavior change wheel, HIV, Quality improvement, Implementation science

## Abstract

Loss to follow-up (LTFU) in high-resolution anoscopy (HRA) programs jeopardizes the procedure’s potential to help prevent anal cancer. We explored quality improvement factors to understand how to address this LTFU. Using the transtheoretical COM-B Model (Capability, Opportunity, Motivation, and Behavior) and a sequential explanatory mixed-methods design, we surveyed and interviewed 13 patients who remained engaged in HIV care but who delayed their HRA monitoring or treatment visits in the same community clinic, and 6 HRA clinicians and medical assistants. Analyses involved descriptive statistics and rapid qualitative analysis. Patients were racially, ethnically, and economically representative of the LTFU population, and were generally experienced with HRA (*Mean* HRA visits = 4.6, *SD* = 2.8, *mdn* = 3). Providers were experienced clinicians and medical assistants (*Mean* years providing HRA = 6.0, *SD* = 2.2). Analyses revealed two primary, related barriers: (A) motivational barriers such as physical pain, discomfort, embarrassment, and anxiety; which were largely borne from (B) opportunity barriers such as difficulties with scheduling, inconsistent after-care (particularly for pain and discomfort), anxiety-inducing exam rooms and equipment, and internalized and anticipated stigma. Capability barriers, such as limited health literacy about HRA, were less common and, like motivational barriers, linked to opportunity barriers. Participants recommended potential facilitators, including easier scheduling, standardization of pain management and after-care services, and examination room modifications to reduce anxiety. To retain HRA patients in community settings, interventions should address social and physical opportunity barriers that strongly determine motivational and capability barriers. Improving convenience, standardizing pain management, and introducing stigma interventions specific to HRA, could alleviate both motivational and capability barriers.

## Resumen

La pérdida de seguimiento (LTFU) en los programas de anoscopia de alta resolución (HRA) pone en peligro el potencial del procedimiento para ayudar a prevenir el cáncer anal. Exploramos factores de mejora de la calidad para comprender cómo abordar este LTFU. Utilizando el modelo COM-B transteórico (Capacidad, Oportunidad, Motivación y Comportamiento) y un diseño de métodos mixtos explicativos secuenciales, encuestamos y entrevistamos a 13 pacientes que permanecieron involucrados en la atención del VIH pero que retrasaron sus visitas de seguimiento o tratamiento de la HRA en la misma clínica comunitaria y 6 médicos y asistentes médicos de la HRA. Los análisis involucraron estadísticas descriptivas y análisis cualitativo rápido. Los pacientes eran representativos de la población LTFU en cuanto a raza, etnia, y estatus económico. En general, tenían experiencia con HRA (visitas HRA media = 4,6, DE = 2,8, mdn = 3). Los proveedores eran médicos y asistentes médicos con experiencia (promedio de años proporcionando HRA = 6,0, DE = 2,2). Los análisis revelaron dos barreras principales relacionadas: (A) barreras motivacionales como el dolor físico, la incomodidad, la vergüenza y la ansiedad; que se debieron en gran medida a (B) barreras de oportunidad, como dificultades con la programación, cuidados posteriores inconsistentes (particularmente para el dolor y el malestar), salas de examen y equipos que inducen ansiedad, y estigma internalizado y anticipado. Las barreras a la capacidad, como la limitada alfabetización sanitaria sobre la HRA, fueron menos comunes y, al igual que las barreras motivacionales, estaban vinculadas a las barreras de oportunidades. Los participantes recomendaron posibles facilitadores, incluida una programación más sencilla, la estandarización del manejo del dolor y los servicios de cuidados posteriores, y modificaciones en la sala de examen para reducir la ansiedad. Para retener a los pacientes de HRA en entornos comunitarios, las intervenciones deben abordar las barreras de oportunidades sociales y físicas que determinan fuertemente las barreras motivacionales y de capacidad. Mejorar la conveniencia, estandarizar el manejo del dolor e introducir intervenciones de estigma específicas para la HRA podría aliviar las barreras tanto motivacionales como de capacidad.

## Introduction

The incidence of anal cancer has increased almost two-fold over the last 50 years, and the disease has attracted more attention as the medical field seeks to understand how to prevent anal cancer among those at most risk [1, 2]. More than 80% of anal cancers are caused by infection with specific genotypes of human papillomavirus (HPV), which can progress from anal neoplasia to high-grade squamous intraepithelial lesions (HSIL), the precursor to squamous cell carcinoma of the anus [2]. This progression is highly pronounced among people with HIV (PWH), who experience elevated risk for the development of anal cancer [3]. For this reason, practice and policy organizations in the US recommend that PWH who are diagnosed with anal neoplasia be additionally screened for HSIL via high resolution anoscopy (HRA) [1, 4–9]. HRA-directed biopsy allows providers to identify, treat and monitor HSIL before it can progress to cancer [9]. The ANCHOR Study (Anal Cancer–HSIL Outcomes Research Study), a multiyear randomized controlled trial, recently proved the efficacy of treatment to prevent anal cancer [10]. Its findings are expected to allow stakeholders to coalesce around national guidelines, recommending HRA as standard of care for anal cancer prevention among PWH [11].

The prevention of anal cancer among PWH will require their ongoing engagement in HRA, yet HRA programs that operate without the benefit of research infrastructure and incentivization report notably high losses to follow-up after the procedure. In one large health system in California, fifty-percent of patients did not return after their initial HRA visit [12]. Two New York City HRA programs, in distinct medical systems, found that 42% of patients with HSIL did not return for their follow-up HRA visits [13, 14]. In a similar cohort study at Johns Hopkins, only 26% of eligible patients underwent HRA [15] and, in a Boston sample, only 32% of patients returned for follow-up HRA within the clinic’s recommended 6-month period [16]. As healthcare systems adopt HRA as standard of care [11], we will need to better understand the causes of this poor retention as well as identify potential solutions, if HRA is to reach its potential to contribute to the prevention of anal cancer.

Determinants of HRA loss to follow-up remain understudied, but are likely to include procedure-specific factors [17–20] as well as social and structural factors [14]. In one of the New York HRA programs cited above, multivariable analyses indicated that HIV virologic control as well as social determinants like racial group and insurance type were associated with loss to follow-up [14]. However, further assessment of the causes of loss to follow-up are warranted, given that the sample included PWH who were not only lost to HRA but also to HIV primary care, making it difficult to ascertain HRA-specific determinants. In one qualitative study of barriers to HRA adherence conducted among men who have sex with men (MSM) who were not necessarily lost to follow-up, stigma, scheduling difficulties, and physical discomfort were primary barriers [17]. Other factors, both during and after HRA, may also influence patients’ decision-making about their return for a follow-up visit [18–20], like fear of anal cancer [18] and physical discomfort experienced during the procedure [19]. The physical and psychological impacts of HSIL on quality of life have been well documented [20] but not yet fully assessed as determinants of loss to follow-up specifically for HRA. Findings about colposcopy may indicate additional potential determinants for loss to follow-up in HRA, because cervical cancer shares a common pathogen with anal cancer and involves a preventative procedure that is similar to HRA. Reasons for poor retention in colposcopy are likewise not well documented, but include gaps in patient knowledge, fear of cancer, pain, and, for gender minorities, gender dysphoria [21, 22].

To better understand why patients do not return for HRA and how to facilitate their re-engagement, we explored barriers and facilitators to HRA retention by isolating HRA-specific determinants among patients retained in HIV primary care but at least two months overdue for HRA within the same healthcare organization. We applied the transtheoretical COM-B Model (Capability, Opportunity, Motivation, Behaviour) [23, 24], which has been applied to other medical services [25] and forms the basis for intervention development using the Behaviour Change Wheel (BCW) [24]. The BCW approach allows for the identification of prominent determinants of a behavior as well as potential change strategies and mechanisms of action [24]. Through this COM-B analysis, we hope to elucidate strategies to facilitate patient engagement in HRA.

## Materials and Methods

To explore barriers and facilitators related to retention in HRA, we conducted a sequential explanatory mixed-methods study using an online survey and hour-long in-depth interviews with HRA patients, clinicians and medical assistants at a federally qualified health clinic in New York City. The Institutional Review Boards of the New York State Psychiatric Institute and of Albert Einstein College of Medicine approved all procedures.

## Methods

### Recruitment

To be eligible, patients needed to be: (1) overdue (i.e., more than 2 months past diagnosis) for either treatment of HSIL or for monitoring after treatment of HSIL; (2) 35 years or older; and (3) able to read, write, and speak in English or Spanish. Patients were excluded if treatment had been delayed solely due to the COVID-19 pandemic. HRA providers, whether HRA clinicians or ancillary HRA staff (i.e., medical assistants), were eligible to participate if they were 18 years or older and able to read, write, and speak in English or Spanish.

Recruitment of patients was initiated by the clinic’s Clinical Director of Anal Health (WD), who requested that colleagues give study palm cards with a QR code, URL and research contact information to eligible patients during their primary care and HRA visits. Clinical staff also conducted outreach via phone calls to patients who were lost to follow-up. Patients and HRA providers interested in study participation could screen for initial eligibility online and then enter their contact information or leave a voicemail or text message for research staff. Recruitment occurred over 9 months, between June 2022 and February 2023. Informed consent was conducted online, with the option of live video conferencing, after which clinic staff verified patient eligibility via chart review. Participants were then sent a demographic and psychosocial survey hosted by REDCap.

The study team conducted purposeful sampling for patient interviews to maximize variability in reasons for loss to follow-up and to ensure representativeness of the patient population. A priori criteria included length of time overdue for HRA treatment or monitoring, gender diversity, racial/ethnic diversity, age, time since HIV diagnosis, HRA-experienced versus relatively HRA-naive, borough of service engagement, HRA provider, and stated reasons for loss to follow-up.

Twenty-eight patients provided sufficient information for initial screening. Of these, 3 were ineligible either through online or telephone screening; 1 did not consent; 6 consented but were deemed ineligible based on chart review; and 1 was eligible by chart review but did not complete the online survey. Among the remaining 18 patients, we purposefully sampled 13 for interviews to ensure participation of people of color, those who could reflect on the current HRA program, and those who were more significantly lost to follow-up for HRA. Specifically, we excluded four participants because their reasons for loss to follow-up were not directly related to HRA (e.g., being sick, forgetting, oversleeping, legal issues); and one additional participant who was overdue by just over 2 months but who otherwise regularly attended HRA visits as scheduled. All 6 providers who were approached consented and were interviewed.

Interviews were conducted via HIPAA-compliant Zoom Pro by two trained interviewers (RG and BH), in Spanish or English based on participant preference, after which participants were compensated $100. The research team produced transcripts via HIPAA-compliant Amazon Web Services, then redacted identifying information. 

### Data Collection

#### Quantitative Assessment

##### Sociodemographics

Both patients and providers were asked to report their *age*, *gender identity*, *sexual orientation*, *racial* and *ethnic identification*, *education*, and *income*. Patients were additionally asked about their *relationship status*, *sexual position preference*, *HIV status*, *number of HRA visits*, *use of HRA at other clinics*, and *“outness” related to gender identity*, *sexual orientation* and *HIV*. Providers were additionally asked about the *number of HRA procedures* they had conducted and the *number of years conducting HRA*.

##### Barriers to HRA Retention

The online survey included barriers to HRA retention, based on literature and consultation with clinic staff, and was organized by *Physical and emotional reasons* (e.g., “Physical pain or discomfort”, “Worried about bleeding”) and *Physical setting and social setting reasons* (e.g., “Difficulty scheduling”), corresponding to capability, opportunity and motivation from the COM-B Model.

#### Qualitative Assessment

The study team developed an in-depth interview guide, based on COM-B constructs, to assess barriers to retention and existing and potential strategies to facilitate retention. Both patients and providers were also presented with and asked to explicate their individually-endorsed survey responses about barriers. We additionally asked providers how the site’s involvement in the research study may have enabled quality improvement during the study period.

### Data Analysis

For survey data, we conducted descriptive statistics. For interview data, we conducted rapid qualitative analyses [26, 27] using debriefing forms, data templates and a data matrix. Shortly after each interview, the interviewer completed a debriefing form to capture memorable qualitative data. The debriefing form consisted of retention barriers, existing facilitators and potential facilitators categorized based on COM-B constructs.

The principal investigator (BAK) reviewed the debriefing form together with each interviewer to clarify questions about COM-B categorization before the interviewer passed the form to the second coder. The second coder read the transcript, identified notable quotations and added missing barriers and facilitators. The second coder also coded more nuanced aspects of capability, opportunity and motivation: *physical* and *psychological capability* (e.g., physical strength, skill or stamina; and knowledge, memory, and attention); *physical* and *social opportunity* (e.g. time, location, materials, services performed; and social norms and cues, stigma); and *automatic* and *reflective motivation* (e.g., desires, impulses and inhibitions, automatic reactions, emotions; and roles, identity, goals) [24]. The principal investigator then reviewed and forwarded the template with comments for the two coders to reconcile any discrepancies, consulting the principal investigator as needed. Once templates were reconciled, raw data were transferred to an Excel matrix with barriers, existing facilitators, and potential facilitators grouped under columns of COM-B constructs. Coders then independently summarized themes within each COM-B construct to develop COM-B-specific themes, and presented their findings for discussion and revision during consensus meetings with the principal investigator. Subsequently, the team derived themes across COM-B constructs to articulate holistic themes for barriers and existing and potential facilitators.

## Results

### Sample Characteristics

As seen in Table 1, participants were experienced with HRA. Patients reported an average of 4.6 HRA visits (*SD* = 2.8; *mdn* = 3 visits; range 1–9 visits). One patient reported 1 visit, three reported 2 visits, three reported 3 visits, one reported 4 visits, one reported 5 visits, one reported 8 visits and two reported 9 visits; one additional patient reported more than 1 visit but did not specify the exact number. Patients also varied in the amount of time overdue for a follow-up HRA visit (*mdn* = 9 months, *range* 2–6 months to 9 years). Based on chart review, four patients were overdue by 3–5 months, two by 9–10 months, one by 1.5 years, two by 4 years, and one by nine years. An additional patient was overdue by 2–6 months and another was overdue by more than 3 months (NB: each of these latter patients were categorized by their lower limit to determine the sample’s median time overdue). HRA providers, including both clinicians and medical assistants, reported performing HRA for an average of 6 years (*SD* = 2.2; range 3–9 years), with half performing between 101 and 500 procedures and one-third performing more procedures. Patients were racially, ethnically, and economically representative of the clinic’s lost to follow-up population. Most were middle-aged and identified as cisgender male, people of color, and gay or bisexual. The majority also reported being single and sexually receptive or versatile. All reported viral suppression. Nearly a quarter opted to be interviewed in Spanish.

**Table 1:** Demographic characteristics of HRA patients and providers (*N* = 19)

	*n* (%)	
	Patients	Practitioners
	*n* = 13	*n* = 6
Age in years (*M*, *SD*)*	47.2 (9.4)	41.8 (5.9)
35–54	9 (69.2)	5 (83.3)
55 or over	4 (30.1)	–
Gender Identity		
Cisgender male	11 (84.6)	3 (50.0)
Cisgender female	0 (0)	2 (33.3)
Transgender male	0 (0)	1 (16.7)
Transgender female	1 (7.7)	0 (0)
Nonbinary	0 (0)	0 (0)
Transgender	1 (7.7)	0 (0)
“Out” about gender identity^‡^	8 (61.5)	–
Sexual orientation		
Gay	8 (61.5)	2 (33.3)
Bisexual	3 (23.1)	0 (0)
Queer	1 (7.7)	2 (33.3)
Heterosexual	1 (7.7)	2 (33.3)
“Out” about sexual orientation^†^	6 (46.2)	–
Race and ethnicity (not mutually exclusive)		
Latinx (of any race)	4 (30.8)	3 (50.0)
Asian or Asian American	2 (15.4)	0 (0)
Black or African American	4 (30.8)	1 (16.7)
White or Caucasian (non-Latinx)	3 (23.1)	2 (33.3)
Bi- or multiracial, Additional	1 (7.7)	1 (16.7)
Education		
< 2-year College Degree	9 (69.2)	3 (50.0)
4-year College Degree	3 (23.1)	0 (0)
Masters or Doctoral Degree	1 (7.7)	3 (50.0)
Income		
Below $29,999	7 (53.8)	–
$30,000–$59,999	4 (30.8)	–
$60,000–or more	2 (15.4)	–
Relationship Status		
Single	9 (69.2)	–
Casually dating several people	2 (15.4)	–
Boyfriend/girlfriend/partner/lover	2 (15.4)	–
Sexual position preference		
‘Bottoming’ (receptive)	7 (53.8)	–
‘Topping’ (insertive)	1 (7.7)	–
Versatile’ (both)'	5 (38.5)	–
HIV status		
Diagnosed with HIV > 2 years ago	13 (100.0)	–
Virally suppressed	13 (100.0)	–
“Out” about HIV	4 (30.8)	–
Number of HRA visits (*M*, *SD; mdn* = 3, *range* 1–9)	4.6 (2.8)	–
Received/performed HRA at another clinic	3 (23.1)	0 (0)
Time overdue for HRA (*mdn* = 9 months, *range 2–6 months to 9 years*)**		
Overdue by 2–6 months	6 (46.2)	–
Overdue by 6 months–2 years	3 (23.1)	–
Overdue by 2 years–4 years	2 (15.4)	
Overdue by more than 4 years	2 (15.4)	
Years providing HRA procedure (*M*, *SD*)	–	6 (2.2)
Total HRA procedures performed in lifetime***	–	
101–500	–	3 (50.0)
501–1000	–	1 (16.7)
More than 1000	–	1 (16.7)

*n = 5 and n = 12 because one patient and one provider did not report age, except for the patient who reported categorical age during eligibility screening

^†^n = 11 because two patients did not report

^‡^n = 12 because two patients did not report

**Time overdue for HRA was based on chart review, not self-report

***n = 5 because one provider reported “Do not know or not sure”

### Barriers, Existing Facilitators and Potential Facilitators for Retention in HRA

Barriers reported in the online survey are presented in Table 2 and all barriers and facilitators from the rapid qualitative analysis are presented in Table 3 as a color-coded heatmap. Table 4, which we explicate below, synthesizes only the most salient quantitative and qualitative data into six barrier themes alongside existing and potential facilitators. We organized themes according to patient-, provider-, and system-levels (e.g., patient-level physical discomfort; system-level clinic processes) as well as constructs within the COM-B Model (e.g., reflective motivation; physical opportunity). Existing and potential facilitators in Table 4 are allocated to more than one barrier if participants suggested a cross-cutting impact. To protect confidentiality, demographics do not accompany illustrative quotations.

**Table 2:** Survey-reported barriers to HRA treatment and monitoring visits endorsed by patients and providers (*N* = 19)

	*n* (%)	
	Patients	Providers
	*n* = 13	*n* = 6
Physical and emotional barriers		
Physical pain or discomfort	8 (61.5)	6 (100.0)
Felt embarrassed	7 (53.8)	6 (100.0)
Felt afraid of cancer	5 (38.5)	3 (50.0)
Felt anxiety	5 (38.5)	6 (100.0)
Afraid of physical damage to asshole	4 (30.8)	4 (66.7)
Felt shame	4 (30.8)	4 (66.7)
Mental/emotional pain or discomfort	4 (30.8)	4 (66.7)
Worried about bleeding	4 (30.8)	3 (50.0)
Overwhelmed when it comes to health issues	3 (23.1)	3 (50.0)
Punishment for bottoming	3 (23.1)	2 (33.3)
Punishment for contracting HIV	2 (15.4)	2 (33.3)
Felt NOT medically necessary	1 (7.7)	3 (50.0)
Triggered gender dysphoria	0 (0)	1 (16.7)
Triggered trauma	0 (0)	2 (33.3)
Felt afraid of something else (text entry)^§^	0 (0)	1 (16.7)
Another reason^¶^	2 (15.4)	1 (16.7)
Physical setting and social setting barriers		
Difficulty scheduling	6 (46.2)	6 (100.0)
Not being able to have sex after the procedure	4 (30.8)	5 (83.3)
Inability to perform tasks/work from discomfort	3 (23.1)	4 (66.7)
The COVID-19 pandemic	2 (15.4)	4 (66.7)
The lack of bedside manner by the provider	2 (15.4)	1 (16.7)
Didn’t like the procedure	2 (15.4)	6 (100.0)
Too many people in the examination room	1 (7.7)	2 (33.3)
Lack of privacy (eg disrobing, exposure)	1 (7.7)	1 (16.7)
Pooped or farted during the procedure	1 (7.7)	0 (0)
Lost income due to inability to work	0 (0)	3 (50.0)
Negative financial impact	0 (0)	1 (16.7)
The wait time at the clinic	0 (0)	2 (33.3)
The identity of the clinical staff	0 (0)	1 (16.7)
The clinic felt chaotic	0 (0)	0 (0)
Didn't like the feel of the clinic	0 (0)	0 (0)
Didn't understand why having the procedure	0 (0)	3 (50)
Didn't know needed to return for another visit	0 (0)	2 (33.3)
Language barrier	0 (0)	1 (16.7)
Inappropriate language from provider	0 (0)	0 (0)
Misgendered	0 (0)	0 (0)
Another reason^#^	0 (0)	1 (16.7)

^†^n = 11 because two patients did not report

^‡^n = 12 because two patients did not report

Text-entry responses below are summarized

^§^Provider: patients can feel ashamed about not being “clean down there”

^¶^Patients: the use of dull/old tools caused excessive bleeding and the need to use a taxi; another surgery was a higher priority and I did not want to feel badly around my birthday; Provider: some patients need to perform anal sex work so delay their visits

^#^Provider: normally the room is more relaxing, but unwelcoming exam rooms can cause discomfort

**Table 3:**
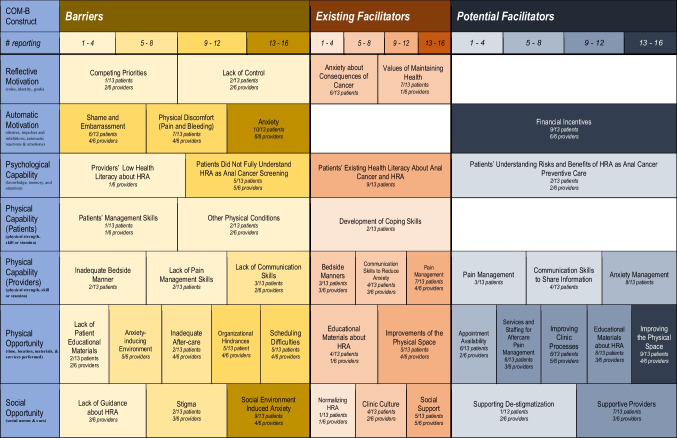
Barriers, existing facilitators and potential facilitators based on qualitative analyses using the COM-B Model (Capability, Opportunity, Motivation and Behavior)

**Table 4:** Mixed-method analysis of HRA retention barriers and facilitators, allocated by patient-, provider-, and system-levels

Themes	Barriers	Existing Facilitators			Potential Facilitators		
Patient-level	Across levels	Patient-level	Provider-level	System-level	Patient-level	Provider-level	System-level
Theme 1. Pain and Discomfort during Recovery Competed with Other Priorities		Values of Maintaining Health			Financial Incentives	Pain Management Skills among Providers	Services and Staffing for Post-Procedure Pain Management
Theme 2. Anxiety Interfered with Engagement	Patient-level Anxiety Interfered with HRA HRA Equipment and Exam Rooms Induced Anxiety The Clinic’s Social Environment Induced Anxiety		Provider Communication Skills to Reduce Anxiety Seeing the Same Supportive Providers	Improvements to the Clinic Space	Financial Incentives	Practical Strategies for Anxiety Management Social Support to Ease Patients’ Anxiety and Discomfort Pain Management Skills among Providers Communication Skills to Lessen Anxiety	Further Improvements to the Physical Space Services and Staffing for Post-Procedure Pain Management
Theme 3. Stigma alongside Emotions of Shame and Embarrassment Deterred Engagement in HRA							
Theme 4. Patients Not Fully Understanding HRA as Anal Cancer Screening Contributed to Loss to Follow-up		Existing Health Literacy about Anal Cancer and HRA				Communication Skills to Lessen Anxiety	Educational Materials about HRA
Provider-Level	Across Levels	Patient-Level	Provider-Level	System-Level	Patient-Level	Provider-Level	System-Level
Theme 5. Communicating about HRA Can Be Difficult						Communication Skills to Lessen Anxiety	Educational Materials about HRA
System-Level	Across Levels	Patient-Level	Provider-Level	System-Level	Patient-Level	Provider-Level	System-Level
Theme 6. System-level Clinic Processes Inhibited Patient Engagement in HRA	Limited Scheduling Organizational Hindrances						Appointment Availability Improving Clinic Processes

### Theme 1 Barrier. Pain and Discomfort during Recovery Competed with Other Priorities (Motivation)

In the survey, most patients (62%) and all providers identified physical pain or discomfort as a barrier to HRA retention. They endorsed that being unable to have sex (30.8% of patients; 83.3% of providers) and to perform daily tasks or work due to pain and discomfort (23.1% of patients; 66.7% of providers) discouraged follow-up visits because of the anticipation of future discomfort. In interviews, patients (38%) revealed that the physical impact of HRA on their daily life activities such as exercise, sex, and work, along with worsening discomfort during the recovery process, also deterred them from seeking further treatment. This was highly endorsed by clinicians as well (67%). One clinician explained:

It’s hard for some folks to take time off from sex because they’re doing sex work and they're making money from having sex and they don’t want, y’know, to have to take time off or because people try to find the right time, like, ‘Oh, my partner is going to be out of town,’ or ‘This is the time when I can take a break from sex,’ and it’s hard to find that time. – Practitioner

#### Existing Facilitator for Theme 1 Barrier

*Values of Maintaining Health *(*Reflective Motivation*) Most patients (86%) reported in interviews that deciding to take responsibility for their health helped them to prioritize HRA visits, a theme supported by a few providers (33%). According to one patient: “I think if anything it makes me feel a little bit like I’m being more responsible if I do go in for the procedure.”

#### Potential Facilitators for Theme 1 Barrier

*Financial Incentives *(*Motivation*) Most patients (69%) and all providers thought that financial incentives would improve HRA retention by mitigating some of the opportunity costs of a visit (e.g., pain, bleeding in public, taking time off from sex work).

*Pain Management Skills Among Providers *(*Capability*) A few patients (23%) recommended that providers should communicate more skillfully and clearly about pain, prescribe pain medications, check in on patients post-procedure, routinely give the same post-procedure instructions, and instruct patients on dietary restrictions and products to manage constipation and other sources of discomfort. One provider (17%) corroborated this need:

Some providers will categorically and consistently give two weeks of opioids, which is way too much, but some people will give a lot of opioids. Some people will give small doses. Some people will give none and just say over the counter. Sometimes … we will give a lidocaine ointment, like a topical, and say to use that with Tylenol and ibuprofen. … I think that the way that we communicate as HRA providers with the patients about the options may not be consistent. –Practitioner

*Services and Staffing for Post-procedure Pain Management (Opportunity) In interviews, p*atients (46%) thought that after-care pain management could be improved by consistently providing: transportation service when bleeding is excessive; information about how to deal with pain and bleeding; and products such as numbing cream, laxatives, and analgesics. Half of healthcare workers also reported that there should be routine follow-up post-procedure pain management, including prescription of pain medication to all patients and a direct point of contact such as a dedicated nurse for patients to follow-up as needed. Providers reported that with more staff trained on HRA they could more directly answer HRA-related questions and respond more quickly with patients’ results.

### Theme 2 Barriers. Anxiety Interfered with Engagement (Motivation)

*Patient-Level Anxiety Interfered with HRA *(*Motivation*) In surveys, about 40% of patients and all practitioners identified anxiety as a barrier, with slightly fewer endorsing specific sources of anxiety (i.e., fear of cancer, worry about bleeding, feeling overwhelmed by health issues). In interviews, patients (77%) expressed anxiety regarding anticipated discomfort (e.g., pain, bleeding) and existential concerns (e.g., addiction to pain medication, cancer). Practitioners (100%) also reported that concerns about pain, precancerous lesions, cancer, and uncertainty about HRA hindered return visits. Patients’ anxiety also affected providers’ ability to perform thorough examinations, biopsies, and treatment because anxiety elevated muscle tone during visits, constricting inspection of the anal canal.

*HRA Equipment and Exam Rooms Induced Anxiety *(*Physical Opportunity*) In interviews, nearly all providers (83%) acknowledged that aspects of HRA equipment and the clinical environment induced anxiety among patients. These included dull biopsy equipment, an uncomfortable exam table (without a proper footrest for HRA), lack of cushions, cramped and impersonal office-like rooms, and inconsistent use of aromatherapy. Only one patient (17%) identified medical equipment as a barrier that contributed to anxiety, but his negative experience deterred his return:

The first time when they did the biopsy, it hurt and the doctor literally told me that the tool wasn’t working right, that it was dull and old. [The] doctor told me this and I was bleeding so much I would practically call it a hemorrhaging and I had to take a taxi home. I couldn't even take the subway because I bled through whatever bandages they gave me and it was still going through my clothes. It was very embarrassing, uncomfortable, mortifying, you name it. And then the second time I went, they told me they hadn’t replaced the tool and they’re like, ‘Don’t worry, we’ll be extra careful.’ But it’s the same shitty, dull tool. And I was like what kind of cheap, budget, bargain basement shit. It’s so upsetting. So that’s why I never went back because I couldn’t trust them to do the procedure, you know, in a proper fashion, using outdated equipment. The doctor is literally telling me it’s old, outdated, and dull. How horrible and scary is that when they’re going, you know, in a very sensitive private area like that? –Patient

*The Clinic’s Social Environment Induced Anxiety *(*Social Opportunity*) In surveys, few participants endorsed crowded rooms or lack of privacy as reasons for not returning for HRA. However, in interviews, 62% of patients and 67% of providers described how the social environment could induce anxiety in ways that discouraged return visits. This included exposure to different medical assistants at each visit, the presence of a female medical assistant for a male patient, too many people in the exam room, a lack of privacy and discretion by the front desk staff, and losing familiar HRA providers when they left the clinic. Patients described their discomfort in interviews:

I just wasn’t prepared when a female nurse came in to assist. I was a little uncomfortable and I couldn’t relax the way I wanted to. –Patient

I do get embarrassed about too many people being in the room. I think at one point there was like three people. I wish they could just be one or two. … I’m too embarrassed. –Patient

The medical assistant was one of the people that takes my blood [during my HIV care visits] and we talk. And he was there. So that was kind of like, ‘Okay, now he’s there helping a doctor, you know, biopsy me naked. –Patient

A few patients were deterred by social interactions, including feeling uncomfortable when a provider and medical assistant laughed without the patient understanding the joke, and, for one patient, a provider’s casual clothes and painted nails.

#### Existing Facilitators for Theme 2 Barriers

*Provider Communication Skills to Reduce Anxiety (Capability) All providers considered communication skills to reduce anxiety essential and a* majority of patients (69%) praised providers’ confidence, use of a gentle and friendly demeanor, and ability to ease patient anxiety with small talk. Additional skills included connecting with patients, avoiding using the word ‘cancer’, not pushing patients into the procedure, not triggering trauma, and being reliable and consistent in their care. They reported that providers have generally communicated well about the procedure, its risks, and the interpretation of results. Communication about pain was a prominent topic. Patients liked when providers candidly discussed expected pain levels, provided guidance on pain management techniques, referred anxious patients to other clinics where HRA is conducted under anesthesia, offered contact information for post-procedure assistance, and checked in with patients after a difficult procedure.

*Seeing the Same Supportive Providers (Social Opportunity)* Patients (62%) reported, and providers supported (83%), that the friendliness and diversity of the clinic, receiving encouragement from their primary care providers, and having a consistent HRA provider motivated return visits for HRA. Patients described how a relationship with a specific provider made their return to care easier:

[I]t has to do with the provider. That the initial provider in [year redacted] made [me] comfortable enough that I was willing to come back and see him again. And that comfortability is why we came back. –Patient

I am very comfortable with [my doctor]. …. It makes me feel relaxed. It makes me feel like, okay, I’m seeing the same person each time I come so therefore I’m not seeing different faces and thinking that people are judging me. –Patient

*Improvements to the Clinic Space (Physical Opportunity)* Patients (38%) reported that a hygienic smell, clean bathrooms, and modern glass doors signaled a modern and organized physical space, particularly in one of the organization’s satellite locations. Healthcare workers (67%) likewise reported beneficial environmental changes to increase patient comfort, including offering anxiolytics prior to the procedure, adding aromatherapy and background music, maintaining optimal room temperature, showing camera images on a monitor, and migrating to a more precise ablation method (i.e., infra-red coagulation to hyfrecation).

#### Potential Facilitators for Theme 2 Barriers

*Further Improvements to the Physical Space (Physical Opportunity)* Most patients (69%) and providers (67%) recommended additional modifications to the physical space to further reduce anxiety and thereby improve the experience of HRA. These included a comfortable place to put one’s legs on the exam table, a video screen to choose whether or not to view the procedure in real-time, a bigger space, relaxing music, dimmed lights, consistent use of aromatherapy, and colorful interior design. Healthcare workers reported similar suggestions and advocated for renovation of the main clinic in particular.

*Practical Strategies for Anxiety Management (Capability)* Though not suggested by providers, most patients (62%) reported that providers could further mitigate patient anxiety by consistently demonstrating compassion and a good bedside manner, treating the patient as a whole person, gauging a patient’s level of discomfort, responding with levity, and maintaining a well-organized HRA visit. Participants also recommended breathing, stretching and yoga exercises to make the environment more ‘Zen’. A simple exchange could have a powerful effect:

A good conversation is something that can eliminate the anxiety ... Making the patient feel comfortable, laugh, give them just something to make them get off that pyramid of being paranoid to just come down to the plateau where like, ‘Okay, my mind is relaxed and once my mind is relaxed, my body [can] just kind of fall in place.’ –Patient

*Social Support to Ease Patients’ Anxiety and Discomfort (Social Opportunity)* To motivate return visits, patients (54%) recommended standards for social support: keeping their familiar HRA provider; pre-procedure counseling to get to know their provider prior to HRA; a male medical assistant or a warning if a female assistant would be present; Spanish-speaking staff; and more discretion about HRA when checking in at the front desk. Half of providers also suggested greater social support to ease patient anxiety.

*Communication Skills to Lessen Anxiety (Capability) Some* patients (31%) reported that providers could give direct and better explanations of the procedure, risks, results, and information in general, including how HSIL affects sexual partners and why a medical assistant is needed in the room to help them feel less anxious about HRA visits.

See Theme 1 for additional cross-cutting Potential Facilitators “Pain Management Skills among Providers” and “Services and Staffing for *Post-Procedure* Pain Management”.

### Theme 3 Barrier. Stigma alongside Emotions of Shame and Embarrassment Deterred Engagement in HRA (Social Opportunity and Automatic Motivation)

In the survey, few patients associated HRA with punishment for engaging in sex or contracting HIV, and only one-third mentioned shame as a barrier. However, in interviews, most patients (62%) and all providers discussed that internalized and anticipated stigma alongside shame and embarrassment affected HRA retention. Some felt that screening was like a punishment for “lifestyle choices” leading to HIV, HPV and, eventually, HSIL.

This is what you get when you have HIV, which is truly not the case, actually, but it does not help, but you can’t help to feel that way. And how others are viewing you in that particular moment, in that room... you’re a spectacle at that point. Are they laughing at you? Are they thinking how gross you may be? ‘This is what he deserves.’ Do they truly care about you or is it just a job for them to be nosy and to get their own personal kicks out of you? –Patient

Patients expressed shame and embarrassment related to exposing an intimate body part, feeling unclean because they could not douche before HRA, and passing gas during the procedure. Two shared incidents of feeling embarrassed when encountering an HRA provider outside the clinic and bleeding through clothing in public, both of which discouraged their return to the HRA clinic. Healthcare workers acknowledged that HRA could trigger patients’ past experiences of trauma and stigma, such as “bottom shaming.” They highlighted the emotional vulnerability associated with medical examination of a body part used for sex and the physical and emotional discomfort caused by positioning oneself on the table for HRA. One clinician mentioned how a patient’s use of humor expressed an underlying feeling of embarrassment and vulnerability.

Well, sometimes people, they are embarrassed. You know, like a comment that I’ll get sometimes is, ‘You could have at least bought me dinner first.’ So this is using humor but also bringing light to the fact that like, yeah, you’re medicalizing this part of my body that’s often used for sex. And I think that that can be uncomfortable for people. They can feel vulnerable. –Practitioner

No existing or potential facilitators were reported for Theme 3.

### Theme 4 Barrier. Patients Not Fully Understanding HRA as Anal Cancer Screening Contributed to Loss to Follow-up (Psychological Capability)

In surveys, half of providers thought patients did not understand why they were having HRA, and nearly a quarter of patients reported having not understood the need to return for monitoring or treatment visits. One patient and three clinicians indicated that patients felt the procedure was not medically necessary. In interviews, patients (38%) and providers (83%) revealed a lack of understanding about the purpose of HRA and what to expect before, during, and after, that reduced motivation to return for follow-up visits. Some patients misinterpreted the offer for screening as discrimination based on being gay or having engaged in anal sex, instead of understanding HRA as a standard of care for anal neoplasia among PWH. Misunderstandings about the function of HRA could interfere with attendance:

Sometimes I get the random patient that will say, ‘Well, I don't have a history of colon cancer.’ Like a misunderstanding that this is not what we’re looking at, you know. Or, ‘I've had a colonoscopy already.’ –Practitioner

#### Existing Facilitator for Theme 4 Barrier

*Existing Health Literacy About Anal Cancer and HRA (Psychological Capability)* A majority of patients (69%) reported that understanding anal cancer prevention facilitated their return for monitoring visits. This knowledge included understanding that HRA is part of a larger process for maintaining one’s sexual health and overall health; knowing how to prepare one’s body for HRA; knowing how to position one’s body during the procedure; and knowing about after care for pain and bleeding. Being aware of the risks linked naturally to motivate preventive behavior for one patient:

It is highly recommended to see if there is cancer or not, because it may be fine right now, but not well tomorrow or in two months. Because cancer is treacherous, it’s like diabetes. Right now I’m fine, I’ve already been diagnosed, so that’s very important to be sure of our body. –Practitioner

#### Potential Facilitator for Theme 4 Barrier

*Educational Materials About HRA (Physical Opportunity)* Most patients (62%) and half of providers reported that more patient-facing educational materials about HRA, in addition to providers’ explanations during the procedure, would help them stay engaged in HRA care. They recommended flyers, a video monitor, brief videos or an ad campaign to educate patients about the benefits and risks of HRA; treatment options; and what to expect before, during and after the procedure (including other patients’ experiences and pain management). Healthcare workers echoed these recommendations.

See Theme 2 for additional cross-cutting Potential Facilitator “Communication Skills to Lessen Anxiety”.

### Theme 5 Barrier. Communicating About HRA Can Be Difficult (Capability)

Few survey responses indicated that providers lacked bedside manners. However, in interviews, 54% of patients and 33% of providers noted that communication about HRA can be a barrier to retention. Two patients reported feeling pressured into the procedure even though they did not feel ready, which made them less likely to return for future visits. One reported that, a decade ago, his HRA provider acted cold, rude, and annoyed. The patient decided never to return for HRA:

I think the bedside manner is the most important thing that I kind of felt… he was just doing what he was trained and supposed to do, and I kind of felt dehumanized for that. I was just, like it was just another examination to him, you know? So, it was uncomfortable on that level. –Patient

Patients also reported that misinformation from a provider (e.g., saying an indeterminate test result was positive; scaring the patient; giving too little or too much information) discouraged their return for follow-up. Providers reported that primary care providers do not always communicate effectively about HRA, and that HRA providers themselves can exaggerate risks, degrading trust when patients discover more accurate information.

See Theme 2 for cross-cutting Potential Facilitator “Communication Skills to Lessen Anxiety” and Theme 3 for cross-cutting Potential Facilitator “Educational Materials about HRA”.

### Theme 6 Barriers. System-Level Clinic Processes Inhibited Patient Engagement in HRA (Opportunity)

*Limited Scheduling (Physical Opportunity).* In surveys, nearly half of patients and all providers noted scheduling as a barrier to HRA retention, corroborated by interviews (38% and 67%, respectively). Inconveniences that exacerbated pressure on scheduling included a scarcity of HRA providers, limited appointment slots, and a lack of flexibility to schedule HRA in the afternoon or on weekends. A provider noted that scheduling difficulties could inhibit patient motivation, because patients preferred continuity with the same provider to minimize discomfort during the procedure:

*I think there’s about two, maybe three providers here who do that procedure and they [patients] tend to only do the procedures with their primary, you know, person. So if [provider name] is the only person who does it then they can only see [provider name], they just can’t jump from [provider name] to a different provider. It has to be like the same provider. –Practitioner*

*Additional Organizational Hindrances (Physical Opportunity)* In interviews, patients (38%) cited clinic disorganization, a strict late policy, limited insurance options that restricted their ability to seek care elsewhere, and delays in obtaining after-care assistance (particularly regarding pain management and how to manage diet and bowel movements). Healthcare workers (83%) echoed these concerns and mentioned additional barriers, including data loss during the transition to a new electronic medical record, workflow friction and fatigue caused by combining urgent care and procedural duties on the same day, the absence of a recall system to remind patients of HRA appointments, a quota imposed on urgent care visits, a shortage of nurses and providers who were interested in learning HRA, and insufficient allocation of human resources for follow-up calls and medical interpreters.

#### Potential Facilitators for Theme 6 Barriers

*Appointment Availability (Physical Opportunity)* In interviews, nearly half of patients (46%) reported that expanding appointments to Friday evenings as well as other weekday afternoons, and allowing for a more generous period of consultation time with the HRA provider for patient education were needed. Healthcare workers (33%) likewise cited a need to expand availability by scheduling afternoon visits and adding more HRA providers.

*Improving Clinic Processes (Physical Opportunity)* In interviews, participants recommended improvements to the clinic flow. Patients (46%) suggested reduced wait times, increasing the number of rooms, and rescheduling appointments within a week rather than a month. They desired an easier system to schedule visits, to receive text reminders for upcoming visits, and to know the purpose of the HRA visit in advance (e.g., check-up vs. treatment) for better preparation. Providers (83%) suggested implementing a recall system for 24-h or 48-h follow-up calls regarding pain management. Other proposed system changes included conducting quality improvement to address repeated cancellations or no-shows for follow-up visits, establishing a dedicated communication channel for HRA patients (e.g., dedicated nurse, dedicated phone number), having a nurse provide pre-procedure education, integrating HRA into a comprehensive preventative and primary care checklist alongside vaccines and labs in collaboration with primary care providers, and separating HRA and urgent care visits on different days to reduce provider burnout.

## Discussion

**Fig. 1:**
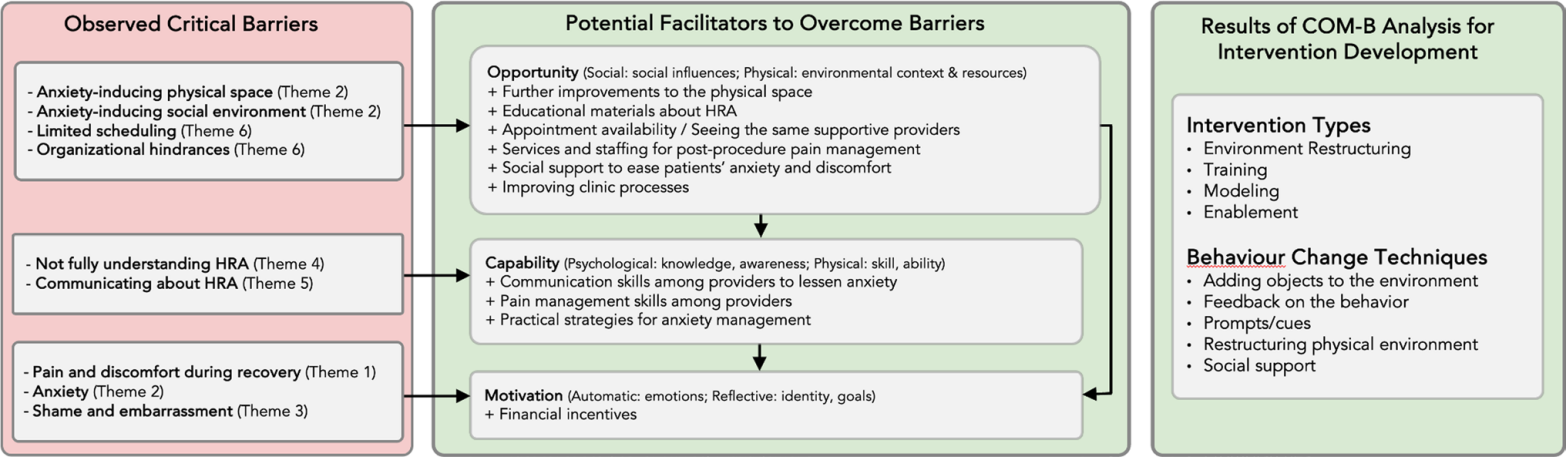
Conceptual model

To our knowledge, our study is one of the first to isolate determinants of HRA retention by recruiting patients engaged in HIV care but lost to follow-up for HRA. As seen in Fig. 1, the most salient barriers were motivational, related to anxiety about shame and embarrassment as well as pain, and, to a lesser extent, capability barriers like knowledge about HRA and anal cancer. These were strongly influenced by opportunity barriers like difficulty scheduling, particularly with the same trusted provider, and a lack of reliable pain management. Existing facilitators that currently re-engage patients in HRA arose primarily from patient desires to protect their health and from the ways that providers ease anxiety and discomfort by attending to the physical spaces where HRA takes place and to how they communicate with patients. The bulk of recommended facilitators involved changes to physical and social opportunity at higher organizational levels to address motivational barriers by improving scheduling, standardizing pain management, and improving other clinic processes.

The finding that patients disliked the HRA procedure because it induced anxiety and physical pain is consistent with reports from other studies [19, 28] and expected given that nerve roots in the anal canal are naturally sensitive to pain [29]. Few remedies to reduce HRA procedural pain have been published, but colposcopy bears similarities to HRA and may provide a corollary to options that reduce pain and anxiety during HRA. For colposcopy, reviews indicate that the use of forced coughing, music, visual distraction, and video of the procedure (but not gentle language) may mitigate pain [30–32]. Additional remedies include injection of medications to anesthetize the area and to constrict blood vessels [33, 34]. However, these remedies have not been examined empirically in the context of HRA, and local anesthesia is already standard of care within the HRA clinic where patients received and delivered services. Music and video distraction could be more standardly offered during HRA, and aligns with recommendations from our participants. In addition to procedural anxiety and pain, we found that physical discomfort following the procedure could last up to a week, further de-motivating patients to return for another HRA visit because of its interference with daily activities and earnings. Standardization of pain management, including setting expectations for pain, offering analgesic prescriptions, and providing services and staffing for after-care should be considered as preemptive measures to retain HRA patients, given the prominence of pain as a deterrent to retention.

Some patients lacked a clear understanding of the purpose of HRA, and therefore questioned the necessity of returning to undergo an uncomfortable procedure, which corroborates a recent finding linking general unawareness of HPV infection and lack of motivation to pursue HRA [35]. In our study, one patient in particular characterized his lack of information as suboptimal informed consent. Other research participants in colposcopy studies have also expressed informational needs about the procedure from their gynecologists, or through other channels including friends, workshops, and pamphlets [21, 36]. The association between a patient’s understanding and their belief of the necessity of medical intervention and the likelihood of adherence is widely established, at least for adherence to medication [37, 38]. Less is known about the relationship between beliefs about necessity and adherence to medical procedures. However, in studies with qualitative data about HRA, beliefs about the necessity of HRA as an anal cancer preventative tool were associated with adherence [17, 35]. All of our patient participants remained retained in HIV care; early investment in the preparation of patients’ knowledge about HRA by their HIV primary care doctors would close these informational gaps, increasing familiarity with the procedure and thereby motivation. Different venues to share information should be implemented, such as handing out pamphlets to supplement discussion and the organization of peer-to-peer support groups to facilitate discussion about HRA.

During interviews, all providers and most patients described stigma as a prominent barrier to returning for HRA. Internalized and anticipated stigma are well-established barriers to HIV care [39], but research about the impact of stigma on HRA retention is nascent and mostly qualitative [17, 35, 40–42]. At least one study has quantified that HIV-related stigma is associated with 46% lowered odds of returning for HRA monitoring [42]. Other studies have found that negative connotations related to HIV/AIDS, HPV, and engagement in anal sex have de-motivated individuals to seek and receive preventative care [40, 43–45]. In our study, patients appreciated the inclusive and diverse organizational culture of the FQHC, which specializes in serving sexual and gender minorities in an urban setting.

This enabling environment of the clinic may contribute to patient retention in HIV care, yet was not sufficient to prevent patient loss to follow up in HRA, which suggests additional needs to address stigma related to HRA itself. Other researchers have recommended increasing consumer awareness of the link between anal HPV and cancer as a means to reduce the deterrent effects of stigma [17], a potential facilitator also mentioned by a small number of participants in our study. However, the effects of educational interventions to reduce stigma related to HPV remains unclear; a study in Nigeria increased HPV knowledge in women but this did not correlate with a change in stigma [46]. Our finding that stigma functions as a salient barrier, with few participants recommending specific remedies, suggests a need to integrate stigma reduction into specific HIV services, as others have recommended [47].

Limitations to our findings include selection bias and social desirability bias. Our recruitment is likely to have biased the inclusion of patients who were willing to re-engage in HRA or, as was the case for at least some, at least willing to discuss their reasons for not re-engaging. Indeed, patients generally had participated in more than four HRA visits (range 2 to 9 visits), an indication that we heard from those who were willing to engage multiple times in HRA prior to disengagement. This selection bias may not reveal determinants of HRA retention among patients with more immediate and more critical perspectives on HRA services. However, this may also indicate that patients do indeed seek to stay in care but lack interventions between HRA visits that could sustain their engagement over the long term. Another limitation is that we assessed determinants only among patients retained in HIV care, allowing us to isolate HRA-specific determinants but likely biasing our results away from social determinants of HIV care that likewise contribute to loss to follow-up in HRA services. Likewise despite protections against a breach in confidentiality, providers and patients may have chosen not to report determinants that they perceived might negatively impact their professional lives or medical care. Even with these sources of bias, we nonetheless heard criticisms of HRA service delivery that, if addressed, could positively benefit at least some patients who delay HRA treatment and monitoring. Additionally, our patient sample, with an average of 4.6 HRA visits, was generally experienced with the procedure. As patients have more opportunity to engage in HRA, missing appointments by 2 months may be due to chance and, for some, inconsequential if they attend soon after the 2-month deadline. We should note, however, that just over half of patients were overdue by 9 or more months; all patients remained engaged in HIV care, but not HRA; and all patients selected HRA-specific reasons for their loss to follow-up. As patients more and less experienced with HRA monitoring and all lost to follow-up for the procedure, their perspectives do indeed contribute to our understanding of potential areas for quality improvement.

In summary, interventions to improve HRA retention in community settings should, based on the COM-B Model, address social and environmental barriers because these aspects of opportunity strongly determine the salience of motivational and capability barriers for patients. Enhancing convenience, standardizing pain management, and enabling both provider- and peer-mediated social support, including education, could alleviate both motivational and capability barriers. These changes could concurrently target stigma that also deters return visits. In sharing their perspectives, both HRA providers and their patients have conveyed HRA-specific challenges as well as organizational remedies to improve the quality of patient-oriented anal health care for PWH.

## Data Availability

Reasonable requests for de-identified participant data may be submitted to the corresponding author at bryan.kutner@einsteinmed.edu.
